# Associations between adolescents’ internalizing problems and well-being: is there a buffering role of boys’ and girls’ relationships with their mothers and fathers?

**DOI:** 10.1186/s12889-021-11920-4

**Published:** 2021-10-16

**Authors:** Chantie Charissa Luijten, Daphne van de Bongardt, Joran Jongerling, Anna Petra Nieboer

**Affiliations:** 1 Department of Socio-Medical Sciences, Erasmus School of Health Policy & Management, Erasmus University Rotterdam, P.O. Box 1738, 3000 DR Rotterdam, The Netherlands; 2 Department of Psychology, Education & Child Studies, Erasmus School of Social and Behavioural Sciences, Erasmus University Rotterdam, P.O. Box 1738, 3000 DR Rotterdam, The Netherlands

**Keywords:** Adolescence, Internalizing problems, Well-being, Parent–adolescent relationship quality, Gender difference

## Abstract

**Background:**

Internalizing mental health problems (i.e., depression and anxiety symptoms) are known to be related negatively to adolescents’ well-being. However, whether this negative association manifests equally in boys and girls, and the potential buffering role of high-quality relationships with mothers and fathers, remain unknown. Thus, the present study was conducted to 1) investigate associations among adolescents’ internalizing problems and mother– and father–adolescent relationship quality, on the one hand, and adolescents’ well-being, on the other hand, 2) explore the buffering role of high-quality mother– and father–adolescent relationships in the association between adolescents’ internalizing problems and well-being, and 3) examine gender differences in these main and buffering effects.

**Methods:**

The analysis sample consisted of 1064 adolescents (53.7% girls; aged 11–17 years) from three secondary schools in the Netherlands. Participants filled out an online questionnaire incorporating the Mental Health Continuum–Short Form to measure well-being, the Revised Child Anxiety and Depression Scale-25 to measure internalizing problems, and the Network of Relationships Inventory to measure mother– and father–adolescent relationship quality. The cross-sectional data were analyzed using path models in R, controlling for age, ethnocultural background, and education level. Multigroup analyses were performed to identify gender differences.

**Results:**

Adolescents with fewer internalizing problems (β = − 0.40, *p* < 0.001) and adolescents with higher-quality relationships with their mothers and fathers reported higher concurrent levels of well-being (β = 0.10 to 0.18, all *p* < 0.01). The quality of mother-adolescent relationships had a significantly larger association with adolescents’ well-being than that of father-adolescent relationship quality. However, relationships with mothers and fathers did not significantly buffer the association between adolescents’ internalizing problems and well-being. Multigroup analyses revealed no difference between boys and girls.

**Conclusions:**

The current study contributes to the understanding of internalizing problems as an important risk factor for adolescents’ well-being, regardless of the quality of relationships with mothers and fathers. The quality of adolescents’ relationships with their parents is associated positively with their well-being, even in the presence of internalizing problems. These findings underline the importance of mothers’ and fathers’ roles in adolescent boys’ and girls’ well-being.

## Background

Adolescence is a challenging developmental period characterized by multiple emotional, physical, and social transformations [1], which may explain the increased prevalence of internalizing symptoms during this period [2]. A large-scale study of the health and well-being of European adolescents (aged 11–16 years) showed that almost one in five adolescents in the Netherlands experiences internalizing problems [3]. Internalizing problems include depression, anxiety, social withdrawal, and somatic or physical problems (e.g., fears, concerns, headaches, and stomachaches) [4, 5]. The most common of these problems among adolescents are anxiety and depression [6].

In recent decades, many empirical studies have shown that adolescents’ internalizing problems are associated with decreased levels of well-being, as these problems negatively affect how adolescents feel about themselves and their way of living [7, 8]. Well-being is defined as a combination of a hedonic conception, focusing on, for instance, happiness, positive emotions, and satisfaction with life, and a eudaimonic conception, comprising good functioning in one’s individual endeavors and social life [9, 10]. Despite consensus in the scientific literature that adolescents with more internalizing problems tend to have lower well-being, knowledge of whether this association manifests equally in boys and girls, and what strengthening or weakening role parents play in it, is limited. The exploration of whether adolescents’ relationships with their mothers and fathers are associated with the well-being of boys and girls, especially those with more internalizing problems, is of great relevance because such problems tend to have chronic, recurring courses and can persist into adulthood [11, 12]. Thus, internalizing problems not only affect adolescents and their family and school environments, but also impose a significant public health burden [13].

Although, the concepts of internalizing problems and well-being are often used interchangeably in the literature, they are not the same. According to the dual-continuum model, mental health problems (e.g., internalizing problems) and well-being are related, yet distinct, continua, rather than opposite ends of a single continuum [14]. According to this model, although the experience of internalizing problems can interfere with well-being, this relationship is not of a one-to-one nature; adolescents with internalizing problems may have high levels of well-being and vice versa. Empirical research has validated the dual-continuum model by identifying these subgroups of young people (i.e., those with high levels of internalizing problems, high levels of well-being and those with low levels of internalizing problems, low levels of well-being) [15, 16] and revealing merely moderate correlations between internalizing problems and well-being (e.g., [17–19]).

In examining the association between adolescents’ internalizing problems and well-being, social determinants, including characteristics of adolescents’ social environments, must be considered in addition to individual characteristics. According to ecological theories of adolescent development (e.g., [20]), relationships with parents are among the most proximal and prominent of adolescents’ social relationships. Adolescents’ perception of the *quality* of their relationships with their parents, which can be characterized by the levels of warmth and conflict [21], are important for their internalizing problems and well-being [22–25]. For instance, adolescents (aged 10–15 years) in the United Kingdom with higher-quality relationships with their parents reported higher well-being than did those with lower-quality relationships [25].

Higher-quality parent–adolescent relationships have been argued theoretically to be resources that can help to reduce or buffer adverse outcomes (e.g., lower well-being) among adolescents with heightened exposure to risk factors (e.g., internalizing problems) [26]. The buffering ability of these relationships has been studied in several contexts (e.g., in examining the link between early sexual activity and adolescents’ well-being [27]), and mixed results have been reported. The potential buffering effect of high-quality parent–adolescent relationships on the association between adolescents’ internalizing problems and well-being has not been evaluated.

In addition, most studies of parent–adolescent relationship quality have not distinguished between relationships with mothers and those with fathers, implying that these relationships are interchangeable [28, 29]. Family systems theory (e.g., [30]) suggests that families consist of several subsystems, including various dyadic family relationships (e.g., mother–child, father–child) that are partly independent and partly interdependent, and as such continuously and reciprocally affect one another. Increasing evidence indicates that mothers and fathers play unique roles in the development of children and adolescents [31–33]. Thus separate examination of the roles of mother– and father–adolescent relationships is important.

In the present study, we aimed to address these research gaps by: 1) investigating the associations among adolescents’ internalizing problems and mother– and father–adolescent relationship quality, on the one hand, and adolescents’ well-being, on the other hand, 2) exploring the buffering role of high-quality mother– and father–adolescent relationships in the association between adolescents’ internalizing problems and well-being, and 3) examining gender differences in these main and buffering effects. We hypothesized that adolescents’ internalizing problems would be associated negatively with their well-being, supporting published findings [7, 8]. In addition, we expected that the quality of mother– and father–adolescent relationships would be related directly and positively to adolescents’ well-being, supporting the theoretical importance of considering maternal and paternal factors in research on adolescents [28, 29].

Various theoretical models describe how relationships with multiple social agents (e.g., mothers and fathers) have simultaneous (i.e., interactive) effects on adolescents’ well-being. For example, according to the compensation model, a high-quality relationship with one parent may buffer the link between an adolescent’s internalizing problems and well-being, compensating the effect of a low-quality relationship with the other parent [34]. According to the additive model, however, adolescents with high-quality relationships with both parents benefit more than those with such a relationship with only one parent in terms of the buffering role of these relationships [34]. Thus, we also exploratively examined three-way interaction effects among adolescents’ internalizing problems and the quality of their relationships with their mothers and fathers.^1^

Research on dyadic family relationships has shown that parent–adolescent relationships differ depending on the gender of both parents and adolescents [35]. Mothers, rather than fathers, tend to remain the primary attachment figures during their sons’ and daughters’ adolescence, contributing more to their development, including their mental health and well-being [31, 36, 37]. Fathers tend to spend more time with their sons than with their daughters from early childhood [38], and the quality of father–adolescent relationships tends to be more important for the development (e.g., self-esteem) of sons relative to daughters [39]. Thus, we hypothesized that high-quality relationships with mothers would be stronger buffers than high-quality relationships with fathers in the association between the internalizing problems and well-being of adolescent boys and girls, and that high-quality father–adolescent relationships would be stronger buffers for boys than for girls.

## Methods

### Participants

Data for this study were collected in the context of a larger project examining socioecological predictors of well-being in adolescents aged 11.0–17.0 years. The initial sample consisted of 1124 adolescents. We excluded participants who reported that they did not have a mother and/or father (*n* = 55) and those with missing data for all variables of interest (*n* = 5). Thus, adolescents whose parents were both alive were included in this study, regardless of family structure and living situation (e.g., living with both parents [77.5%], living with one parent [14.5%], separated parents with co-parenting [7.4%], living with others such as foster parents [0.6%]). Thus, the final sample consisted of 1064 adolescents (53.7% girls) aged 11.0–17.0 years (mean [M] = 13.7 years, standard deviation [SD] = 1.1 year).

Based on the stratified Dutch secondary education system, most (74.2%) participants were enrolled in higher (i.e., senior general [hoger algemeen voortgezet onderwijs] and pre-university [voorbereidend wetenschappelijk onderwijs]) education and 25.8% were enrolled in lower (i.e., pre-vocational [voorbereidend middelbaar beroepsonderwijs]) education. More than half (57.7%) of the participants had Western ethnocultural backgrounds (i.e., they and their parent[s] were born in Europe, the United States, Canada, Australia, or New Zealand); 42.3% had non-Western backgrounds (i.e., they and/or their parent[s] were born in Africa, the Middle East, Asia, Latin America, or South America). The excluded students (*n* = 60) were significantly older (M = 14.03 years, SD = 1.35 years) than the included participants, with no difference in internalizing problems and well-being. Because of this difference and the documented relevance of age to the central concepts investigated in this study (e.g., [25]), age was included as a control variable.

### Procedure

The participants were recruited from three secondary schools in the Netherlands that provided active informed consent for their students’ participation. Seventh-, eighth-, and ninth-grade students and their parents or guardians received information letters by email describing the study aims and procedure, the right to voluntary participation, and confidentiality of data. Informed consent was taken from the parents/guardians as all participants were minors (under 18 years old). Upon receipt of parental or guardian consent, informed consent from adolescents was obtained separately; the adolescents provided face-to-face consent and were allowed to decline participation or withdraw from the study at any time. In total, 6.2% (*n* = 84) of contacted parents/guardians and 1.0% (*n* = 13) of adolescents used this opportunity to decline participation. The medical ethics committee of Erasmus Medical Centre, Rotterdam, the Netherlands determined that the rules stipulated in the Medical Research Involving Human Subjects Act did not apply to this study, and thus that the present study did not need to be further evaluated by an official medical ethical review board (protocol no. MEC-2018-055).

Participating adolescents filled out online questionnaires in their classrooms during regular school hours. Data collection was supervised by the lead researcher (the first author) and research assistants, who introduced the study and procedure, answered questions, ensured maximum privacy, and guaranteed that the students’ responses were confidential. After filling out the questionnaire, the participants received small, non-financial incentives and a card listing websites with information about topics covered in the questionnaire, as well as contact details of the researchers for questions. After data collection, one iPhone per school and one gift card per class (range, €5–10 across grades) were awarded to participants in a raffle.

### Measures

#### Well-being

We used the Dutch Mental Health Continuum–Short Form (MHC-SF) [14], which has been validated for use with Dutch adolescents [19], to measure well-being. The respondents were instructed to rate 14 items on a six-point scale (0 = never, 5 = every day) to indicate their degrees of emotional well-being (three items; e.g., “How often did you feel happy?”), psychological well-being (six items; e.g., “How often did you feel good at managing the responsibilities of your daily life?”), and social well-being (five items; e.g., “How often did you feel that you had something important to contribute to society?”) in the last month. Mean scores for the 14 items were calculated, with higher scores indicating higher levels of well-being. In our sample, the MHC-SF showed good reliability (Cronbach’s *α* = 0.91).

#### Internalizing problems

The Revised Child Anxiety and Depression Scale-25 [40] is a 25-item inventory that measures symptoms of anxiety (15 items; e.g., “When I have a problem, I get a funny feeling in my stomach”) and depression (10 items; e.g., “I have problems with my appetite”). It was developed for use with children and adolescents aged 8–18 years. The items are ranked on a four-point scale (0 = never, 3 = always). The reliability and validity of the depression and anxiety subscales [41], and the reliability of total scores (sums of the two subscale scores) [40, 42], have been confirmed. We used the total scale to measure internalizing problems, with higher scores indicating more frequent symptoms of these problems (i.e., depression and anxiety). This scale showed good reliability in the present study (Cronbach’s *α* = 0.91).

#### Quality of parent–adolescent relationships

The quality of mother– and father–adolescent relationships was measured using the satisfaction (three items; e.g., “How satisfied are you with the relationship with your mother/father?”) and conflict (three items; e.g., “How much do you and your mother/father argue with each other?”) subscales from the Network of Relationships Inventory [21]. The items are ranked on a six-point scale (1 = none, 6 = the most). Total mother– and father–adolescent relationship quality scores were calculated using all items, with the ranking reversed for the conflict subscale items; higher scores reflect higher overall relationship quality [43]. For our sample, we obtained Cronbach’s *α* values of 0.89 for mothers and 0.90 for fathers.

### Statistical analyses

Descriptive statistics were calculated for internalizing problems, the quality of mother– and father–adolescent relationships, and well-being using SPSS software (version 25; IBM Corporation). Independent-samples *t* tests were performed to compare the mean levels of these variables between boys and girls. We also examined bivariate correlations, with *r* values of 0.10–0.29, 0.30–0.49, and ≥ 0.50 considered to reflect weak, moderate, and strong correlations, respectively [44]. The alpha level was set at 5.0%.

**Fig. 1:**
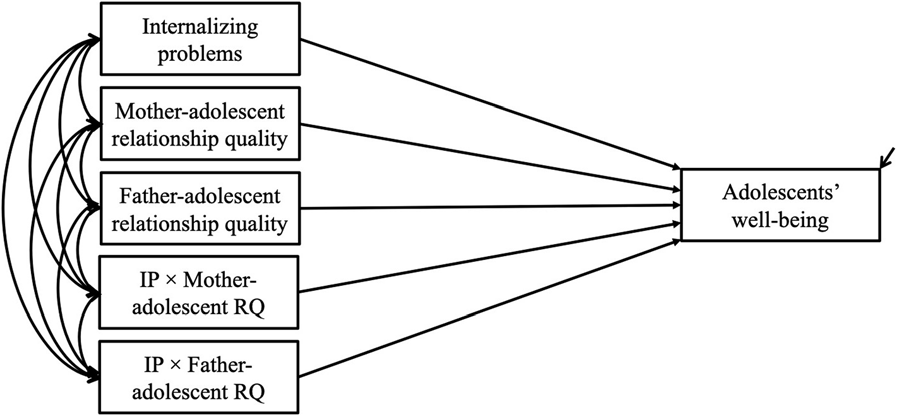
Simplified path diagram of the proposed moderation model. Note. Covariates (i.e., age, ethno-cultural background, and education level) are not depicted in the model. IP = internalizing problems, RQ = relationship quality

To test our hypotheses, we performed structural equation modeling to test path models (see Fig. 1) in R (version 4.0.3; R Core Team) using the lavaan package [45]. As missing value analysis performed with SPSS software (version 25; IBM Corporation) revealed missing values for internalizing problems (0.2%), well-being (0.2%), and mother– (0.4%), and father– (0.7%) adolescent relationship quality, largely because not all participants completed the online questionnaire, all structural equation modeling was performed using full information maximum likelihood estimation [46]. To deal with nonnormality in the data, we used robust maximum likelihood estimation, which corrects for deviations from multivariate normality by computing robust standard errors and adjusted chi-squared values asymptotically equivalent to the Yuan–Bentler test statistic [47, 48]. All continuous independent variables were centered to minimize multicollinearity. In addition to age, ethnocultural background (0 = Western, 1 = non-Western) and education level (0 = low, 1 = high) were included as control variables because of their documented relevance to the central concepts examined in this study [25, 49–52].

To investigate the main associations of internalizing problems and mother– and father–adolescent relationship quality with adolescents’ well-being, we tested four models. Model 1, used to examine the main effect of internalizing problems on well-being, was expanded to include data on mother– and father–adolescent relationship quality separately (models 2 and 3) and together (model 4). Two- and three-way interaction effects of mother– and father–adolescent relationship quality were then examined separately and together in two additional models (internalizing problems × mother–adolescent relationship quality and internalizing problems × father–adolescent relationship quality [model 5], and internalizing problems × mother–adolescent relationship quality × father–adolescent relationship quality [model 6]).

Sample size calculation with Gpower [53] revealed that 176 participants would be required to detect small to medium main effects with 95% power and a type 1 error rate of 5%. The final sample of 1064 adolescents provided sufficient power to test the main effects and two-way interaction effects [54, 55]. The sample required to test three-way interaction effects is less clear. As interaction effects are typically difficult to detect in non-experimental designs and require relatively large samples, the results of the exploratory analysis of three-way interaction effects should be interpreted with caution. If significant interaction effects were detected, stratified analyses were performed to explore group differences in parent–adolescent relationship quality.

To identify differences between boys and girls, multigroup analyses were performed using models 4 and 5. An unconstrained multigroup model, in which each parameter was estimated separately for boys and girls, was compared with constrained models in which intercepts and regressions were fixed separately and together between boys and girls. Significant increases or decreases in model fit were taken to reflect gender differences using the Santorra-Bentler *χ*^*2*^ statistic, root mean square error of approximation (RMSEA), comparative fit index (CFI), and standardised root mean square residual (SRMR). Values of RSMEA < .06, CFI > .95, and SRMR ≤ .08 indicated a good to excellent model fit, whereas RSMEA < .08 and CFI > .90 indicated a satisfactory fit [56, 57].

## Results

### Sample characteristics

Levels of well-being and mother– and father–adolescent relationship quality were significantly higher among boys than among girls, and girls experienced significantly more internalizing problems than did boys (Table 1). Significant correlations among well-being, internalizing problems, and mother– and father–adolescent relationship quality were found for boys and girls (Table 2). Correlations with internalizing problems were negative, whereas those between mother– and father–adolescent relationship quality and well-being were positive. Most correlations were moderate; strong correlations were found between internalizing problems and well-being for girls (*r* = 0.52), and between mother– and father–adolescent relationship quality for boys (*r* = 0.56). The correlation between father–adolescent relationship quality and well-being in girls was weak (*r* = 0.28). Age correlated significantly, but weakly, with all variables of interest for girls, but not boys.

**Table 1:** Sample characteristics

	M (SD***)***		
	Total sample	Girls	Boys
Well-being	3.39 (0.97)	3.27 (0.95)	3.53^a^ (0.99)
Internalizing problems	11.15 (9.04)	13.57^b^ (10.02)	8.32 (6.73)
Mother-adolescent relationship quality	4.87 (0.79)	4.82 (0.84)	4.94^a^ (0.73)
Father-adolescent relationship quality	4.74 (0.95)	4.66 (0.99)	4.84^a^ (0.90)

All differences between girls and boys were significant (*p* < 0.05)

^a^Larger mean scores for boys, ^b^larger mean scores for girls

**Table 2:** Pearson correlations between variables of interest for male and female adolescents

	1	2	3	4	5
1. Well-being	–	−.52***	.35***	.28***	−.13**
2. Internalizing problems	−.47***	–	−.36***	−.30***	.09*
3. Mother-adolescent relationship quality	.40***	−.43***	–	.35***	−.19***
4. Father-adolescent relationship quality	.32***	−.35***	.56***	–	−.19***
5. Age	.03	−.04	−.02	−.09	–

Correlations above the diagonal are for girls, and those below the diagonal are for boys. *p < 0.05, **p < 0.01, ***p < 0.001

### Associations between internalizing problems, mother– and father–adolescent relationship quality, and well-being

Participants’ age, ethnocultural backgrounds, and education level together explained 1.0% of the variance in adolescents’ well-being (*R*^2^ = 0.01, *p* < 0.001), but these covariates were not associated significantly with adolescents’ well-being in models 1–4 (Table 3). In Model 1 internalizing problems and the covariates explained 25.6% of variance in adolescents’ well-being. Internalizing problems were associated negatively with adolescents’ well-being (Table 3). The addition of mother– and father–adolescent relationship quality (models 2 and 3, respectively) explained an additional 3.6% (∆*R*^2^ = 0.04, *p* < 0.001) and 2.0% (∆*R*^2^ = 0.02, *p* < 0.001) of the variance in adolescents’ well-being, respectively. Both of these variables were associated positively with adolescents’ well-being (Table 3). Model 4 including both mother– and father–adolescent relationship quality explained 29.9% of the variance in adolescents’ well-being. Internalizing problems were associated negatively with adolescents’ well-being and the qualities of adolescents’ relationships with their mothers and fathers were associated positively with adolescents’ well-being (Table 3). The association between mother–adolescent relationship quality and well-being was significantly larger than that of father–adolescent relationship quality and well-being (*p* = 0.034). The goodness-of-fit indices of models 1 to 4 revealed good to excellent fit (Table 4).

**Table 3:** Structural equation modeling results for main effects

	Model 1 Base model			Model 2 Mothers only			Model 3 Fathers only			Model 4 Mothers and fathers		
	b	SE	β	b	SE	β	b	SE	β	b	SE	β
Intercept	3.45^***^	0.06	3.55^***^	3.42^***^	0.06	3.51^***^	3.44^***^	0.06	3.54^***^	3.41^***^	0.06	3.51^***^
Age	−0.04	0.03	−0.05	−0.02	0.03	− 0.02	−0.02	0.03	−0.03	− 0.01	0.03	− 0.01
Ethnocultural background	0.02	0.05	0.01	0.03	0.05	0.01	0.03	0.05	0.02	0.03	0.05	0.02
Education level	−0.10	0.06	−0.05	−0.06	0.06	−0.03	− 0.09	0.06	− 0.04	−0.05	0.06	−0.02
IP	−0.05^***^	0.00	−0.50^***^	−0.05^***^	0.00	−0.42^***^	− 0.05^***^	0.00	− 0.45^***^	−0.04^***^	0.00	−0.40^***^
Mother RQ				0.26^***^	0.04	0.21^***^				0.22^***^	0.04	0.18^***^
Father RQ							0.16^***^	0.03	0.16^***^	0.10^**^	0.03	0.10^**^
*R*^*2*^	.26*		.29*			.28*			.30*			

*SE* Standard error, *IP* Internalizing problems, *RQ* Relationship quality. *R*^*2*^ = explained variance in adolescents’ well-being. *p < 0.05, **p < 0.01, ***p < 0.001

**Table 4:** Goodness-of-fit indices for models 1–6

Model	***χ***^***2***^	df	***p***	RMSEA	90% CI RMSEA	CFI	SRMR
Model 1	3.92	1	.048	.05	.00–.11	.99	.01
Model 2	3.93	1	.047	.05	.01–.11	1.00	.01
Model 3	3.98	1	.046	.05	.01–.11	.99	.01
Model 4	3.98	1	.046	.05	.01–.11	1.00	.01
Model 5	3.98	1	.046	.05	.01–.11	1.00	.01
Model 6	4.00	1	.046	.05	.01–.11	1.00	.01

*χ2* = Santorra-Bentler chi-squared test, *df* Degrees of freedom, *CI* Confidence interval, RMSEA Root mean square error of approximation, *CFI* comparative fit index, *SRMR* Standardized root mean square residual. Criteria for good model fit are: RSMEA < .06, CFI > .95, and SRMR ≤ .08

Model 5 with the two two-way interaction effects added to model 4 explained 30.0% of the variance in adolescents’ well-being (*R*^2^ = 0.30, *p* = 0.046) and model 6 including the additional exploratory three-way interaction effect explained 30.0% of this variance (*R*^2^ = 0.30, *p* = 0.046). No significant interaction (buffering) effect was identified. The goodness-of-fit indices of models 5 and 6 revealed good to excellent fit (Table 4).

### Differences between sons and daughters

In the multigroup analyses based on models 4 and 5, the fixing of intercepts revealed significant differences between boys and girls compared with the unconstrained multigroup model (Δ*χ*^*2*^ [7] = 111.70, *p* < 0.001 and Δ*χ*^*2*^ [9] = 123.32, *p* < 0.001), but we found no significant difference between boys and girls after the fixing of regressions. Thus, these analyses revealed no difference in the main associations between boys and girls.

## Discussion

To the best of our knowledge, no studies have investigated whether the well-established negative association between internalizing problems and well-being applies to boys and girls equally, and what potential buffering role parents play in this association. The present study addressed this gap and: 1) investigated the main associations of internalizing problems and the quality of mother– and father–adolescent relationships with adolescents’ well-being, 2) explored the buffering role of high-quality mother– and father–adolescent relationships in the association between internalizing problems and well-being, and 3) examined differences in these main and buffering effects between boys and girls.

Adolescents with more internalizing problems reported lower levels of well-being, in accordance with our expectations based on previous research [7, 8]. The quality of adolescents’ relationships with mothers and fathers had no buffering effect on this association. We found no significant interaction between internalizing problems and mother– or father–adolescent relationship quality in the effect on adolescents’ well-being. Thus, the data do not support the hypothesis that a high-quality relationship with one parent compensates for a low-quality relationship with the other parent (compensation model) or the hypothesis that high-quality relationships with both parents are more beneficial than a single high-quality parental relationship (additive model). Previous research has yielded mixed results regarding the study buffer hypotheses, and analyses that have revealed significant buffering effects of parent–adolescent relationship quality have had small effect sizes [58]. Possible explanations for the lack of buffering effect include the use of different instruments to measure the same constructs and the low variability of risk factor (internalizing problems) scores [59]. Our sample was a general school population of non-clinical adolescents, most of whom had few internalizing problems, good relationships with their parents, and high well-being levels. The results might differ for adolescents with (sub-)clinical levels of internalizing problems and lower-quality relationships with their parents [59]. We thus recommend additional research examining similar hypotheses with other adolescent samples, including (sub-)clinical samples, to determine whether the current findings can be replicated.

Although theory suggests the importance of the buffering effects of high-quality relationships, more empirical support exists for their main effects (for a review, see [58]). Indeed, our results revealed that adolescents with higher-quality relationships with their mothers and fathers reported higher levels of well-being, even after controlling for internalizing problems. We also found that the relation between the quality of mother-adolescent relationships and adolescents’ well-being was significantly stronger than that between father-adolescent relationship quality and adolescents’ well-being. This finding suggests the importance of investigating the effects of both parents on adolescents’ well-being, supporting our expectations based on family systems theory (e.g., [30]) and previous research [23, 60, 61]. Adolescence is characterized by social transformations, among others [1], and research suggests that the relative roles of mothers and fathers change between adolescence and young adulthood [61]. Thus, future research should investigate the unique roles of mothers and fathers with respect to adolescents’ internalizing problems and well-being over time.

We found that girls had more internalizing problems and lower well-being than did boys, in line with previous findings [3, 7, 51]. In contrast to previous research, in which girls reported higher-quality relationships with their mothers and fathers than did boys [62], we found that boys had higher-quality relationships with both parents than did girls. This finding may be explained by girls’ experience of greater changes, such as increased conflict [63], in their relationships with their mothers and fathers, relative to boys. For instance, among adolescents aged 12–18 years in the United States’ New England region, girls perceived stronger increases in alienation from both parents and stronger declines in trust with mothers than did boys [64].

Although we found gender differences in the mean levels of internalizing problems, well-being, and mother–, and father–adolescent relationship quality, structural equation modeling revealed no gender difference in a main or buffering effect on adolescents’ well-being. Fewer internalizing problems and higher-quality relationship with parents were associated significantly with greater well-being among boys and girls. To better understand the roles of parents in the relationship between adolescent boys’ and girls’ internalizing problems and well-being, future research could focus on aspects of parent–adolescent relationships other than quality, such as effects on adolescents’ emotion regulation pathways. The importance of studying parental depressive symptoms has been demonstrated, as these symptoms may interfere with parents’ ability to take care of their children [65]. In response, adolescents may have dissociative experiences and internalizing problems, which in turn lead parents to commit affective communication errors, including contradictory signaling and failure to initiate responsive behavior to their children’s cues. Such maternal and paternal behaviors in the parental dyad may have cumulative negative effects on adolescents’ development [65].

The present study was strengthened by the use of a large, culturally diverse sample of adolescents, distinction between mother– and father–adolescent relationships, and the inclusion of positive and negative aspects of social relationship quality. Our findings contribute to the existing literature and increase our understanding of the negative association between internalizing problems and well-being that applies to boys and girls regardless of the quality of adolescents’ relationships with their mothers or fathers. However, some limitations of this study need to be considered. First, the analysis of cross-sectional questionnaire data does not permit determination of the directionality or causality of associations among variables. In addition, as the prevalence of internalizing problems is known to increase throughout adolescence [6, 66], longitudinal data are required to advance our understanding of individual and longitudinal changes in relationships between adolescents’ internalizing problems and well-being, as well as possible changes in the roles of parents over time. We also recommend additional (longitudinal studies) with larger samples to examine the potential buffering roles of high-quality mother– and father–adolescent relationships on adolescents’ internalizing problems and well-being in greater depth and with sufficient power (to detect three-way interactions).

Second, our results were based on adolescents’ self-reports, and are thus subject to potential response bias. For example, although our participants reported few internalizing problems overall, adolescents with more such problems may have answered questions about the quality of their relationships with their parents and their well-being differently (e.g., more negatively) in comparison with adolescents with no internalizing problems [67].

Third, although this study built on family systems theory [30], we assessed only adolescents’ relationships with their mothers and fathers, and not, for instance, relationships between parents or of adolescents with their siblings. In addition, despite substantial support for the reliability and predictive utility of adolescents’ self-reports about their relationships with their parents [68], future research may benefit from the inclusion of reports from other actors in multiple family subsystems (e.g., parents) to more fully capture adolescents’ relationships or interactions with their family members and effects on their internalizing problems and well-being.

Fourth, although parents are important proximal socializing agents, young people expand their interpersonal networks and place greater emphasis on peer relationships, particularly friendships, in adolescence [69]. Peer relationships have been found to be important to adolescents’ well-being [70]. In addition, theoretical and empirical research has demonstrated the value of considering independent and joint effects of relationships from different contexts (e.g., family members and peers) in the examination of adolescents’ well-being [71, 72]. Thus, future studies of the association between adolescents’ internalizing problems and well-being should include consideration of the role of peer relationships.

Finally, participants in this study were younger than excluded students, which may have influenced our results despite the control for age in our analyses. Also, Dutch adolescents are, on average, among the happiest and most satisfied with their lives of adolescents worldwide [3]. Thus, results might differ in other populations and countries where adolescents do not experience similar levels of well-being. To test the generalizability of our findings, this study should be replicated with adolescents throughout the Netherlands and in other countries.

## Conclusions

In this study, adolescent boys and girls with more internalizing mental health problems reported less well-being, regardless of the quality of their relationships with their mothers and fathers. Nonetheless, higher-quality relationships with both mothers and fathers were related significantly to higher well-being of adolescents, even in the presence of internalizing problems. Higher-quality mother-adolescent relationships were associated with even higher levels of adolescents’ well-being than father-adolescent relationships of similar quality. These findings may contribute to future public health policy making, as adolescents’ internalizing problems and well-being are increasingly being recognized as major priorities [73, 74]. Prevention and intervention programs that aim to enhance the well-being of adolescents with internalizing problems may be improved by the inclusion of strategies to help adolescents achieve or maintain high-quality relationships with their mothers and fathers [20].

## Data Availability

The datasets generated and/or analyzed during the current study are available from the corresponding author on reasonable request.

## Abbreviation

MHC-SF: Mental Health Continuum–Short Form

## Footnotes

1 These results should be interpreted with caution, as the statistical power for three-way interaction effects may be limited.

## References

[1] Steinberg L, Morris AS (2001). Adolescent development. Ann Rev Psychol.

[2] Reitz E, Deković M, Meijer AM (2005). The structure and stability of externalizing and internalizing problem behavior during early adolescence. J Youth Adolesc.

[3] Stevens GWJM, Van Dorsselaer S, Boer M, de Roos S, Duinhof EL, ter Bogt TFM, Van den Eijnden R, Kyper L, Visser D, Vollebergh W, de Looze M. HBSC 2017. Gezondheid en welzijn van jongeren in Nederland [Health and well-being of young people in the Netherlands]. 2018 https://hbsc-nederland.nl/wp-content/uploads/2018/09/Rapport-HBSC-2017.pdf. Accessed 21 Dec 2020.

[4] Merrell KW, Merrel KW (2008). Understanding internalizing problems: depression and anxiety in children and adolescents. Helping students overcome depression and anxiety, second edition: A practical guide.

[5] Plenty S, Östberg V, Almquist YB, Augustine L, Modin B (2014). Psychosocial working conditions: an analysis of emotional symptoms and conduct problems amongst adolescent students. J Adolesc.

[6] Costello EJ, Copeland W, Angold A (2011). Trends in psychopathology across the adolescent years: what changes when children become adolescents, and when adolescents become adults?. J Child Psychol Psychiatry.

[7] Bartels M, Cacioppo JT, Van Beijsterveldt TC, Boomsma DI (2013). Exploring the association between well-being and psychopathology in adolescents. Behav Genet.

[8] Lyons MD, Huebner ES, Hills KJ, Van Horn ML (2013). Mechanisms of change in adolescent life satisfaction: a longitudinal analysis. J Sch Psychol.

[9] Diener E, Diener E (2009). Subjective well-being. The science of well-being.

[10] Gallagher MW, Lopez SJ, Preacher KJ (2009). The hierarchical structure of well-being. J Pers.

[11] Garber J, Weersing VR (2010). Comorbidity of anxiety and depression in youth: implications for treatment and prevention. Clin Psychol (New York).

[12] Scholten WD, Batelaan NM, van Balkom AJ, Penninx BW, Smit JH, van Oppen P (2013). Recurrence of anxiety disorders and its predictors. J Affect Disord.

[13] Gore FM, Bloem PJ, Patton GC, Ferguson J, Joseph V, Coffey C (2011). Global burden of disease in young people aged 10–24 years: a systematic analysis. Lancet.

[14] Keyes CL (2005). Mental illness and/or mental health? Investigating axioms of the complete state model of health. J Consult Clin Psychol.

[15] Greenspoon PJ, Saklofske DH (2001). Toward an integration of subjective well-being and psychopathology. Soc Indic Res.

[16] Suldo SM, Shaffer EJ (2008). Looking beyond psychopathology: the dual-factor model of mental health in youth. Sch Psychol Rev.

[17] Antaramian SP, Huebner ES, Hills KJ, Valois RF (2010). A dual-factor model of mental health: toward a more comprehensive understanding of youth functioning. Am J Orthop.

[18] Haworth CM, Carter K, Eley TC, Plomin R (2017). Understanding the genetic and environmental specificity and overlap between well-being and internalizing symptoms in adolescence. Dev Sci.

[19] Luijten CC, Kuppens S, van de Bongardt D, Nieboer AP (2019). Evaluating the psychometric properties of the mental health continuum-short form (MHC-SF) in Dutch adolescents. Health Qual Life Outcomes.

[20] Bronfenbrenner U (1979). The ecology of human development: experiments by nature and design.

[21] Furman W, Buhrmester D (2009). Methods and measures: the network of relationships inventory: behavioral systems version. Int J Behav Dev.

[22] Hale WW, Nelemans SA, Meeus WH, Branje SJ (2020). A 6-year longitudinal study of adolescents and mothers depression symptoms and their perception of support and conflict. Child Psychiatry Hum Dev.

[23] Chu PS, Saucier DA, Hafner E (2010). Meta-analysis of the relationships between social support and well-being in children and adolescents. J Soc Clin Psychol.

[24] Guo C, Tomson G, Keller C, Söderqvist F (2018). Prevalence and correlates of positive mental health in Chinese adolescents. BMC Public Health.

[25] Yucel D, Yuan ASV (2016). Parents, siblings, or friends? Exploring life satisfaction among early adolescents. App Res Qual Life.

[26] Fergus S, Zimmerman MA (2005). Adolescent resilience: a framework for understanding healthy development in the face of risk. Ann Rev Public Health.

[27] Markham CM, Lormand D, Gloppen KM, Peskin MF, Flores B, Low B, et al. Connectedness as a predictor of sexual and reproductive health outcomes for youth. J Adolesc Health. 2010;46(3):S23–41. 10.1016/j.jadohealth.2009.11.214. 10.1016/j.jadohealth.2009.11.214 20172458

[28] Cabrera NJ, Fitzgerald HE, Bradley RH, Roggman L. The ecology of father-child relationships: an expanded model. J Fam Theory Rev. 2014. 10.1111/jftr.12054.

[29] Flouri E (2010). Fathers’ behaviors and children's psychopathology. Clin Psychol Rev.

[30] Cox MJ, Paley B (1997). Families as systems. Ann Rev Psychol.

[31] Keizer R, Helmerhorst KO, Van Rijn-van Gelderen L (2019). Perceived quality of the mother–adolescent and father–adolescent attachment relationship and adolescents’ self-esteem. J Youth Adolesc.

[32] Lewis C, Lamb ME (2003). Fathers’ influences on children’s development: the evidence from two-parent families. Eur J Psychol Educ.

[33] Silva RNA NAE, Van de Bongardt D, Van de Looij-Jansen P, Wijtzes A, Raat H (2016). Mother– and father–adolescent relationships and early sexual intercourse. Pediatrics.

[34] Zhang S, Baams L, Van de Bongardt D, Dubas JS (2018). Intra-and inter-individual differences in adolescent depressive mood: the role of relationships with parents and friends. J Abnorm Child Psychol.

[35] McHale SM, Crouter AC, Whiteman SD (2003). The family contexts of gender development in childhood and adolescence. Soc Dev.

[36] Branje SJ, Hale WW, Frijns T, Meeus WH (2010). Longitudinal associations between perceived parent-child relationship quality and depressive symptoms in adolescence. J Abnorm Child Psychol.

[37] Rosenthal NL, Kobak R (2010). Assessing adolescents’ attachment hierarchies: differences across developmental periods and associations with individual adaptation. J Res Adolesc.

[38] Pleck JH, Masciadrelli BP, Lamb ME (2004). Paternal involvement by US residential fathers: levels, sources, and consequences. The role of the father in child development.

[39] Song H, Thompson RA, Ferrer E (2009). Attachment and self-evaluation in Chinese adolescents: age and gender differences. J Adolesc.

[40] Ebesutani C, Reise SP, Chorpita BF, Ale C, Regan J, Young J, et al. The revised child anxiety and depression scale-short version: scale reduction via exploratory bifactor modeling of the broad anxiety factor. Psychol Assess. 2012;24(4):833–45. 10.1037/a0027283. 10.1037/a0027283 22329531

[41] Klaufus L, Verlinden E, van der Wal M, Kösters M, Cuijpers P, Chinapaw M (2020). Psychometric evaluation of two short versions of the revised child anxiety and depression scale. BMC Psychiatry.

[42] Piqueras JA, Martín-Vivar M, Sandin B, San Luis C, Pineda D (2017). The revised child anxiety and depression scale: a systematic review and reliability generalization meta-analysis. J Affect Disord.

[43] Van de Bongardt D, De Graaf H, Reitz E, Deković M (2014). Parents as moderators of longitudinal associations between sexual peer norms and Dutch adolescents’ sexual initiation and intention. J Adolesc Health.

[44] Cohen J (1988). Statistical power analysis for the behavioral sciences.

[45] Rosseel Y. Lavaan: An R package for structural equation modeling and more. Version 0.5–12 (BETA). J Stat Softw. 2019; https://users.ugent.be/~yrosseel/lavaan/lavaanIntroduction.pdf. Accessed 10 Jan 2021.

[46] Enders CK, Bandalos DL (2001). The relative performance of full information maximum likelihood estimation for missing data in structural equation models. Struct Equ Modeling.

[47] Sass DA, Schmitt TA, Marsh HW (2014). Evaluating model fit with ordered categorical data within a measurement invariance framework: a comparison of estimators. Struct Equ Modeling.

[48] Yuan KH, Bentler PM (2000). Three likelihood-based methods for mean and covariance structure analysis with nonnormal missing data. Sociol Methodol.

[49] Kriesi I, Buchmann M, Jaberg A. Educational success and adolescents’ well-being in Switzerland. Schweiz Z Soziol. 2012. 10.5167/uzh-68739.

[50] Salmela-Aro K, Tynkkynen L (2010). Trajectories of life satisfaction across the transition to post-compulsory education: do adolescents follow different pathways?. J Youth Adolesc.

[51] Smith NR, Lewis DJ, Fahy A, Eldridge S, Taylor SJC, Moore DG, et al. Individual socio-demographic factors and perceptions of the environment as determinants of inequalities in adolescent physical and psychological health: the Olympic regeneration in East London (ORiEL) study. BMC Public Health. 2015;15(1):150. 10.1186/s12889-015-1459-1. 10.1186/s12889-015-1459-1 PMC4339478 25884502

[52] Vacek KR, Coyle LD, Vera EM (2010). Stress, self-esteem, hope, optimism, and well-being in urban, ethnic minority adolescents. J Multicult Couns Devel.

[53] Faul F, Erdfelder E, Lang AG, Buchner A (2007). G* power 3: a flexible statistical power analysis program for the social, behavioral, and biomedical sciences. Behav Res Methods.

[54] Shieh G (2009). Detecting interaction effects in moderated multiple regression with continuous variables power and sample size considerations. Organ Res Methods.

[55] Shieh G (2010). Sample size determination for confidence intervals of interaction effects in moderated multiple regression with continuous predictor and moderator variables. Behav Res Methods.

[56] Bentler PM, Bonett DG (1980). Significance tests and goodness of fit in the analysis of covariance structures. Psychol Bull.

[57] Hu LT, Bentler PM (1999). Cutoff criteria for fit indexes in covariance structure analysis: conventional criteria versus new alternatives. Struct Equ Modeling.

[58] Lakey B, Orehek E (2011). Relational regulation theory: a new approach to explain the link between perceived social support and mental health. Psychol Rev.

[59] Cohen S, Wills TA (1985). Stress, social support, and the buffering hypothesis. Psychol Bull.

[60] Moak ZB, Agrawal A (2010). The association between perceived interpersonal social support and physical and mental health: results from the National Epidemiological Survey on alcohol and related conditions. J Public Health.

[61] Wang Z, Kouvonen A, Satka M, Julkunen I (2019). Parental social support and adolescent well-being: a cross-sectional study in China. Child Indic Res.

[62] Van Eijck FE, Branje SJ, Hale WW, Meeus WH (2012). Longitudinal associations between perceived parent-adolescent attachment relationship quality and generalized anxiety disorder symptoms in adolescence. J Abnorm Child Psychol.

[63] Simon VA, Furman W (2010). Interparental conflict and adolescents’ romantic relationship conflict. J Res Adolesc.

[64] Ebbert AM, Infurna FJ, Luthar SS (2019). Mapping developmental changes in perceived parent–adolescent relationship quality throughout middle school and high school. Dev Psychopathol.

[65] Vismara L, Sechi C, Lucarelli L. Fathers’ and mothers’ depressive symptoms: internalizing/externalizing problems and dissociative experiences in their adolescent offspring. Curr Psychol. 2019. 10.1007/s12144-019-00566-6.

[66] Bor W, Dean AJ, Najman J, Hayatbakhsh R (2014). Are child and adolescent mental health problems increasing in the 21st century? A systematic review. Aust N Z J Psychiatry.

[67] De Los RA, Goodman KL, Kliewer W, Reid-Quinones K (2008). Whose depression relates to discrepancies? Testing relations between informant characteristics and informant discrepancies from both informants’ perspectives. Psychol Assess.

[68] Metzler CW, Biglan A, Ary DV, Li F (1998). The stability and validity of early adolescents’ reports of parenting constructs. J Fam Psychol.

[69] De Goede IH, Branje SJ, Meeus WH (2009). Developmental changes in adolescents’ perceptions of relationships with their parents. J Youth Adolesc.

[70] Raboteg-Šarić Z, Šakić M (2014). Relations of parenting styles and friendship quality to self-esteem, life satisfaction and happiness in adolescents. App Res Qual Life.

[71] Llorca A, Cristina Richaud M, Malonda E. Parenting, peer relationships, academic self-efficacy, and academic achievement: direct and mediating effects. Front Psychol. 2017;8. 10.3389/fpsyg.2017.02120. 10.3389/fpsyg.2017.02120 PMC5736920 29326615

[72] Sechi C, Vismara L, Lucarelli L (2020). Attachment to parents and peers and adolescent mental health: the mediating role of alexithymia. Comm Ment Health J.

[73] Parkin E, Long R, Gheera M. Children and young people’s mental health: policy, services, funding and education. No. 07196. 2019. https://dera.ioe.ac.uk/30819/1/CBP-7196%20_Redacted.pdf. Accessed 6 Apr 2021.

[74] World Health Organization. Mental health action plan 2013-2020. 2013. https://apps.who.int/iris/bitstream/handle/10665/89966/9789241506021_eng.pdf. Accessed 10 Mar 2021.

